# Supplementary material for: Design of synthetic collagens that assemble into supramolecular banded fibers as a functional biomaterial testbed
Source: Nat Commun. 2022 Nov 9;13:6761. doi: 10.1038/s41467-022-34127-6 (PMC9646729; doi:10.1038/s41467-022-34127-6)
Supplement: Supplementary file 1 — Supplementary Information [file 41467_2022_34127_MOESM1_ESM.pdf]

Supplementary Information for

Design of synthetic collagens that assemble into supramolecular banded fibers as a functional biomaterial testbed

Jinyuan Hu, Junhui Li, Jennifer Jiang, Lingling Wang, Jonathan Roth, Kenneth N. McGuinness, Jean Baum, Wei Dai, Yao Sun, Vikas Nanda, Fei Xu

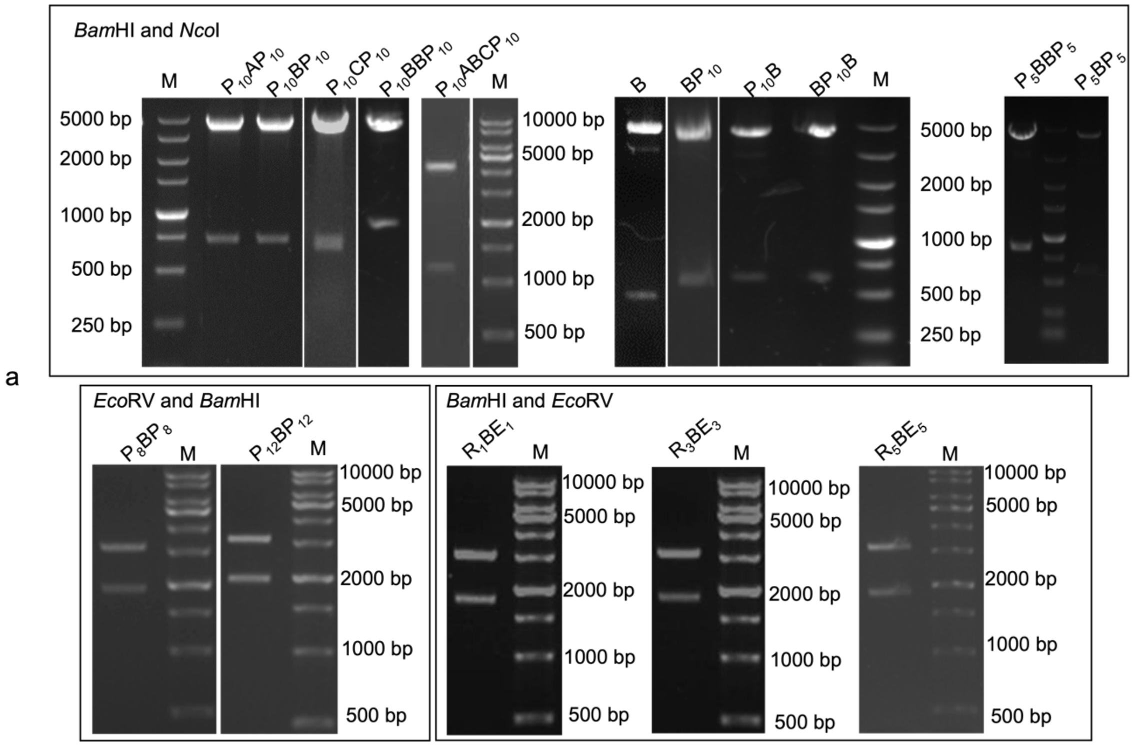

Supplementary Fig. 1 (a) The construction of recombinant collagen plasmids were verified by double enzyme digestion. The recombinant collagen plasmids pColdIII-Tu-*gene* were digested by two restriction endonucleases as labeled（*Bam*HI and *Nco*I, *Eoc*RV and *Bam*HI, *Bam*HI and *Eoc*RV）. All the digested fragments were analyzed by agarose gel electrophoresis and stained with GelRed. Gene names of the synthetic collagens are as labeled in the figure. (*continued on next page*).

b

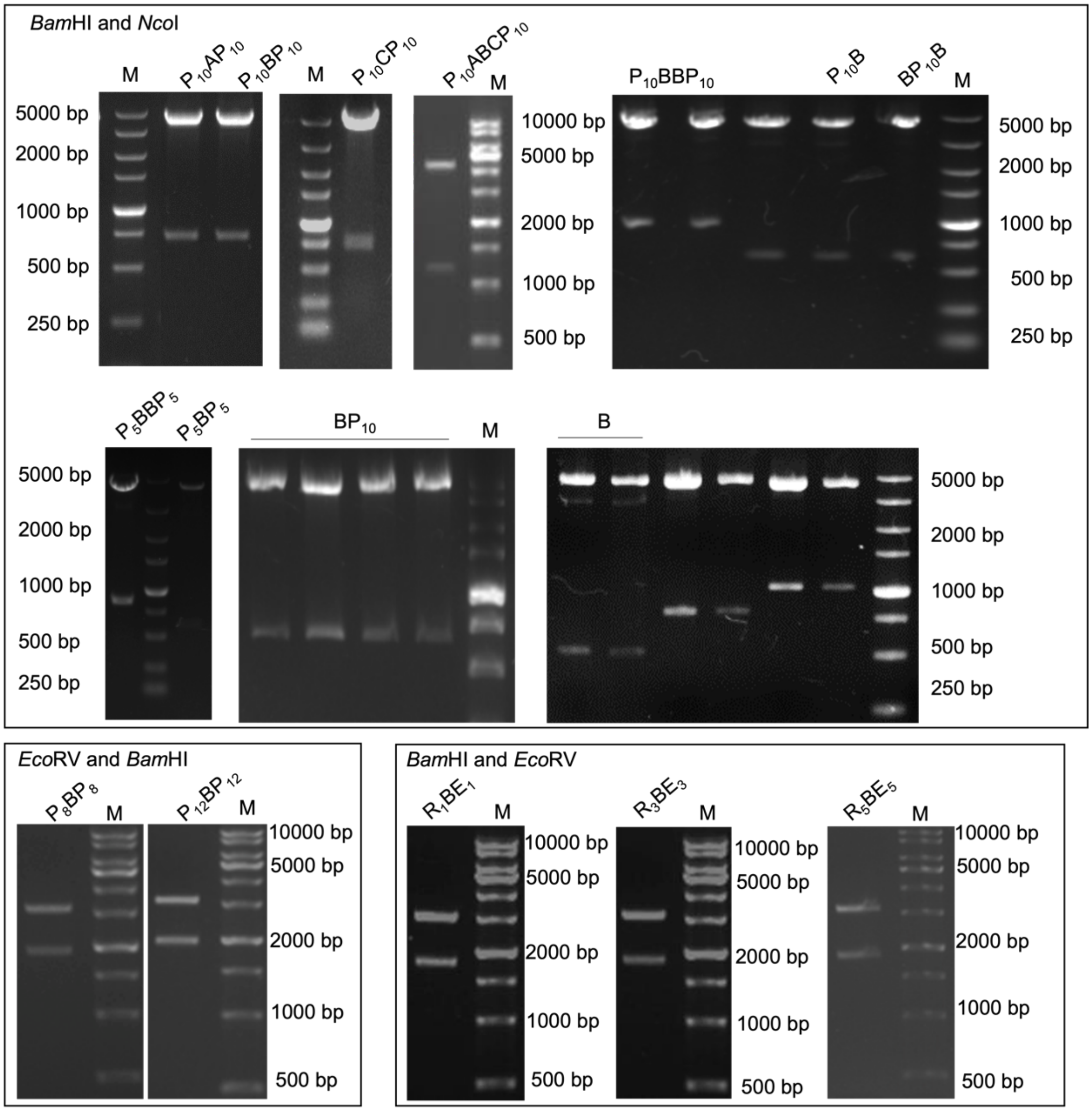

Supplementary Fig. 1 (b) Original gel scans.

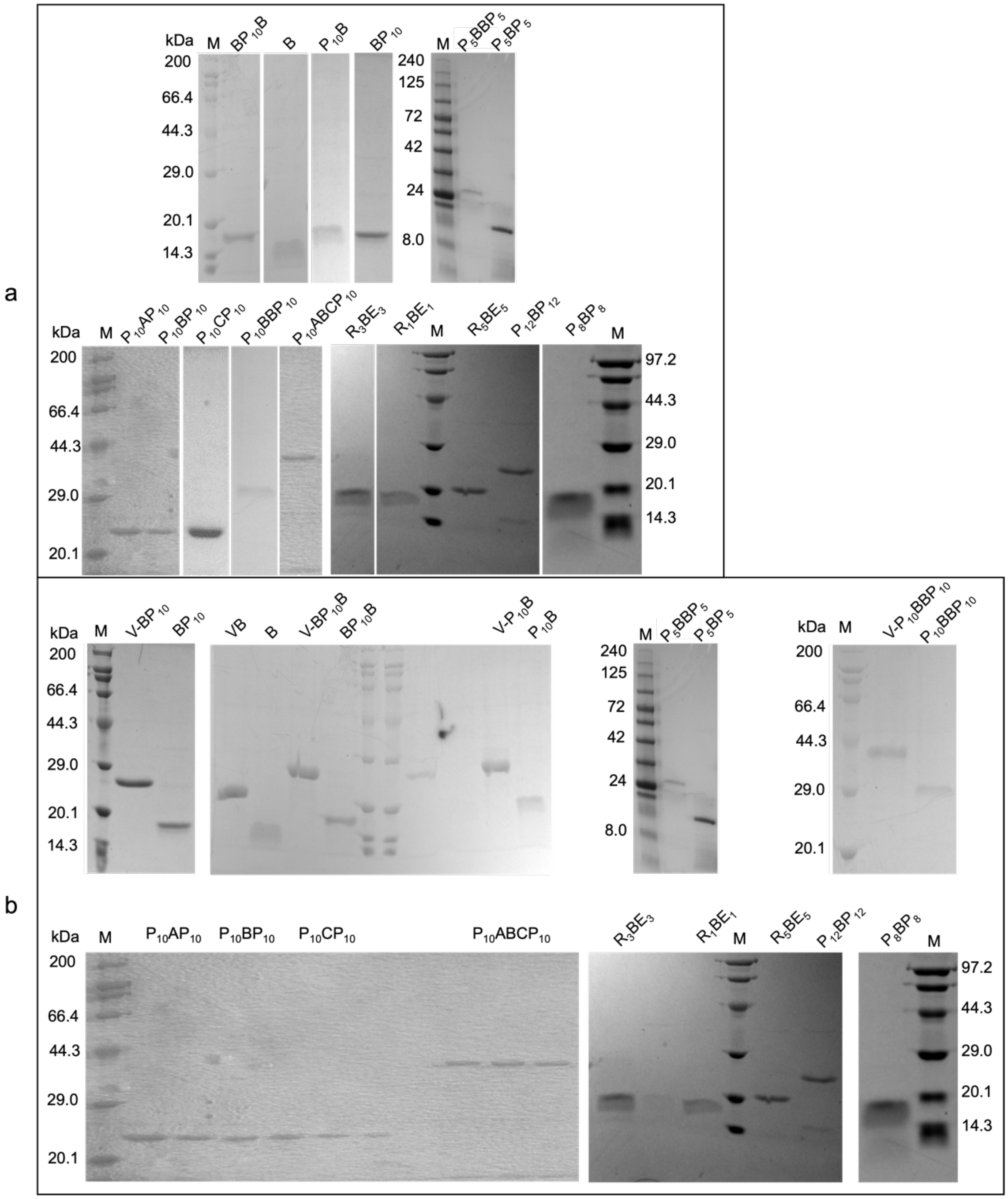

Supplementary Fig. 2 (a) SDS-PAGE of the synthetic collagens after trypsin digestion. The collagen with precollagen V-domain were purified with affinity purification, and digested by trypsin at 25℃ for overnight to remove the V-domain. After dialyzed and freeze-dried, all the synthetic collagens were analysis by SDS-PAGE and stained with Coomassie blue. Names of the synthetic collagens are as labeled in the figure. **(b)** Original gel scans.

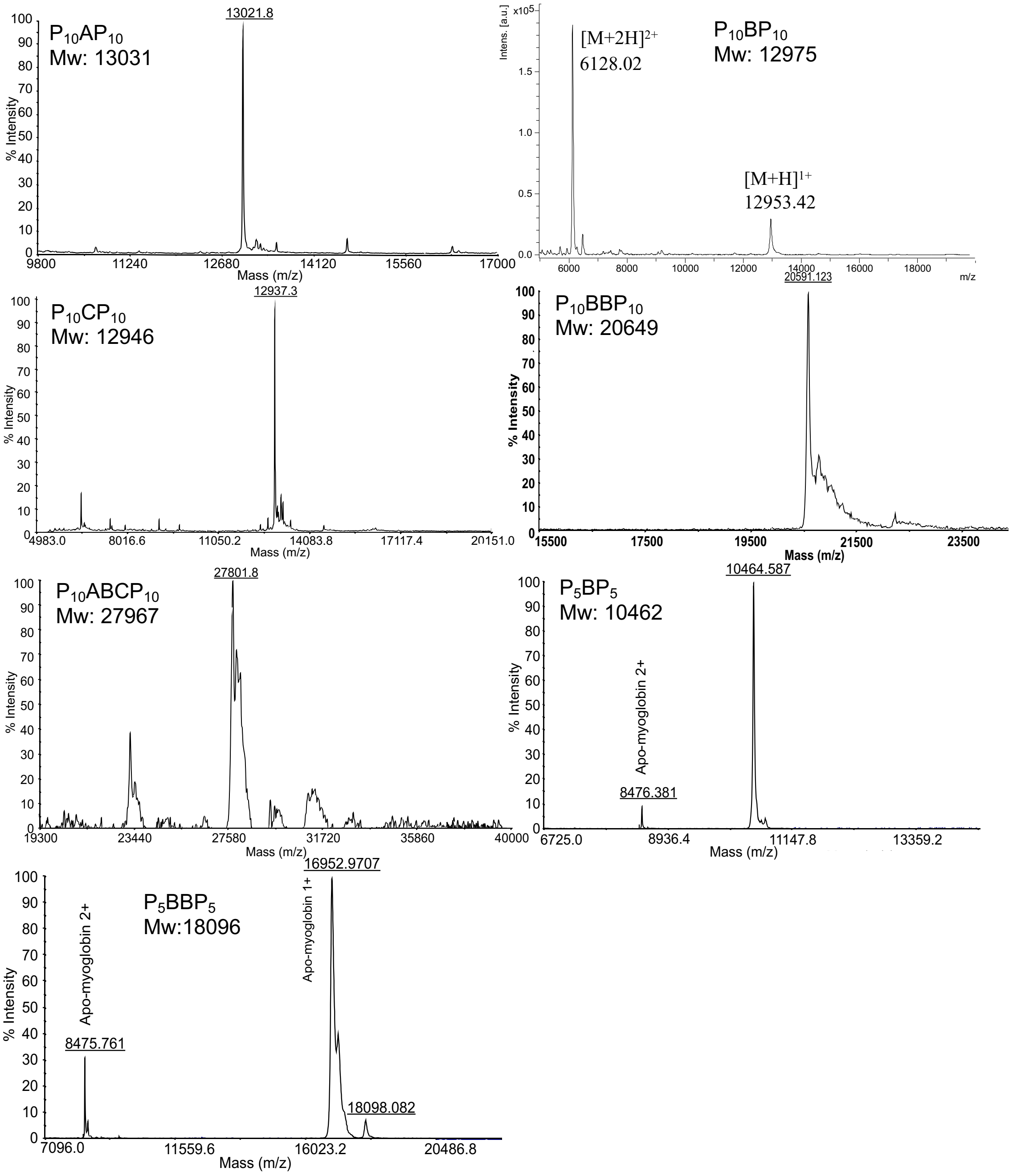

P10AP10
Mw: 13031
13021.8
% Intensity
Mass (m/z)
P10BP10
Mw: 12975
[M+2H]2+
6128.02
[M+H]1+
12953.42
Intens. [a.u.]
m/z
P10CP10
Mw: 12946
12937.3
P10BBP10
Mw: 20649
20591.123
P10ABCP10
Mw: 27967
27801.8
P5BP5
Mw: 10462
10464.587
Apo-myoglobin 2+
8476.381
P5BBP5
Mw:18096
Apo-myoglobin 2+
8475.761
Apo-myoglobin 1+
16952.9707
18098.082

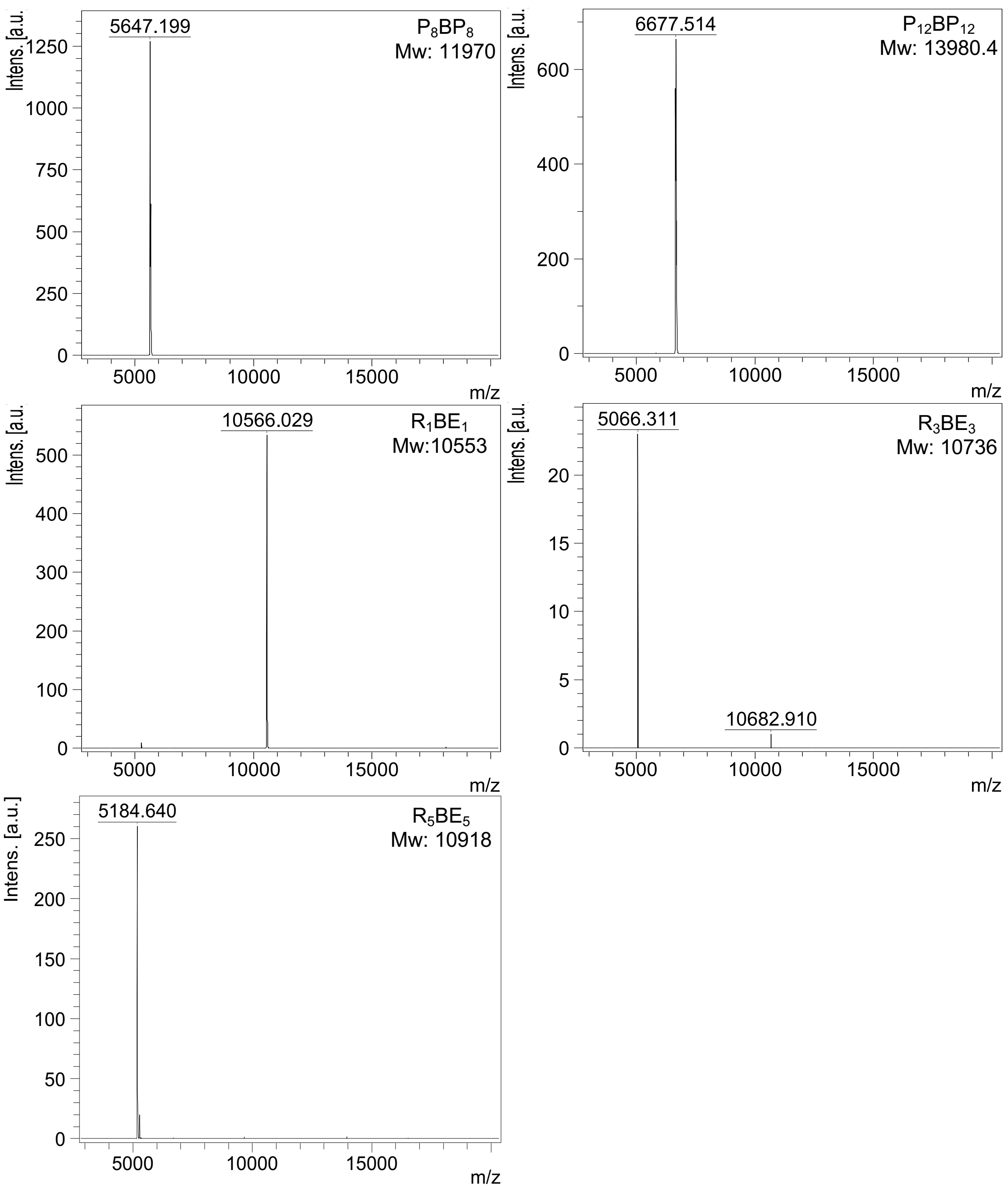
5647.199
P8BP8
Mw: 11970
Intens. [a.u.]
m/z
6677.514
P12BP12
Mw: 13980.4
10566.029
R1BE1
Mw:10553
5066.311
10682.910
R3BE3
Mw: 10736
5184.640
R5BE5
Mw: 10918

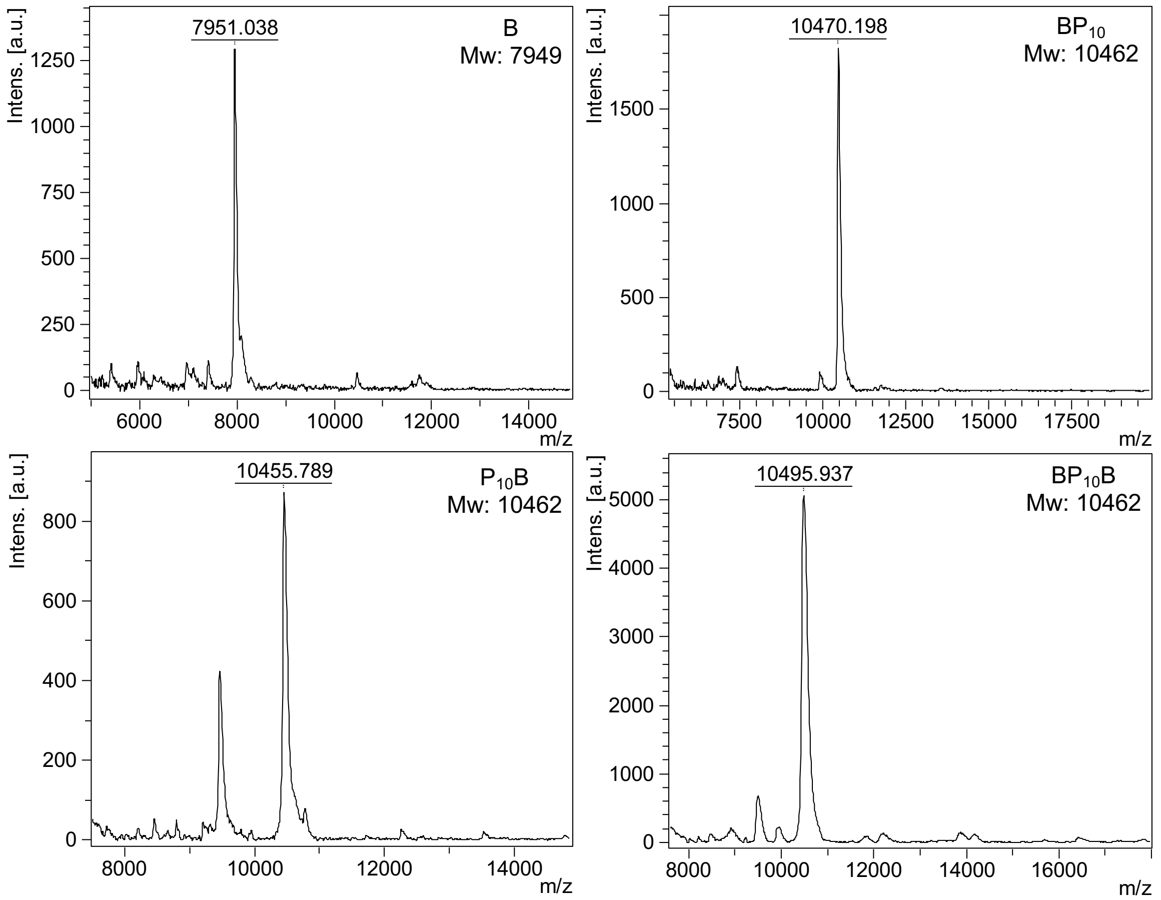

Supplementary Fig. 3. MALDI-TOF mass spectra showing molecular weights of all synthetic collagens. After dialyzed and freeze-dried, all the synthetic collagens were dissolved to 1 mg mL^{-1} in ultrapure water and the molecular weights were analyzed by MALDI-TOF mass spectra. Names of the synthetic collagens are as labeled in the figure.

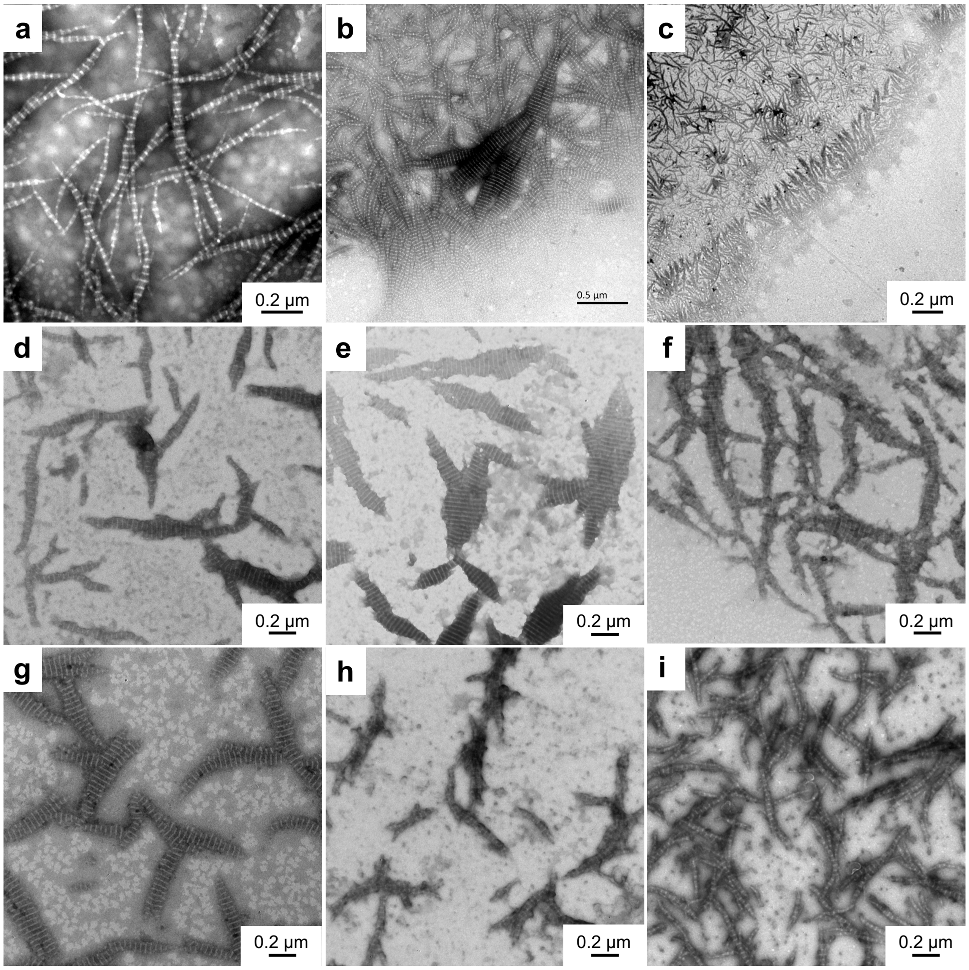

Supplementary Fig. 4. Transmission Electronic Microscopy (TEM) images of $P_{10}BP_{10}$ fibers at various temperatures and salt concentrations. The samples were prepared with 0.5 mM concentration in 10 mM phosphate buffer at pH 7 and incubated at 4°C **(a-c)**, 20°C **(d)**, 30°C **(e)**, and 37°C **(f)** for 3 days. The samples were also prepared under the same condition as Fig. 1f, in a 10 mM phosphate buffer at pH 7 with 50 mM NaCl **(g)**, 100 mM NaCl **(h)**, and 150 mM **(i)**. Before TEM imaging, the samples were negatively stained with 0.75% phosphotungstic acid.

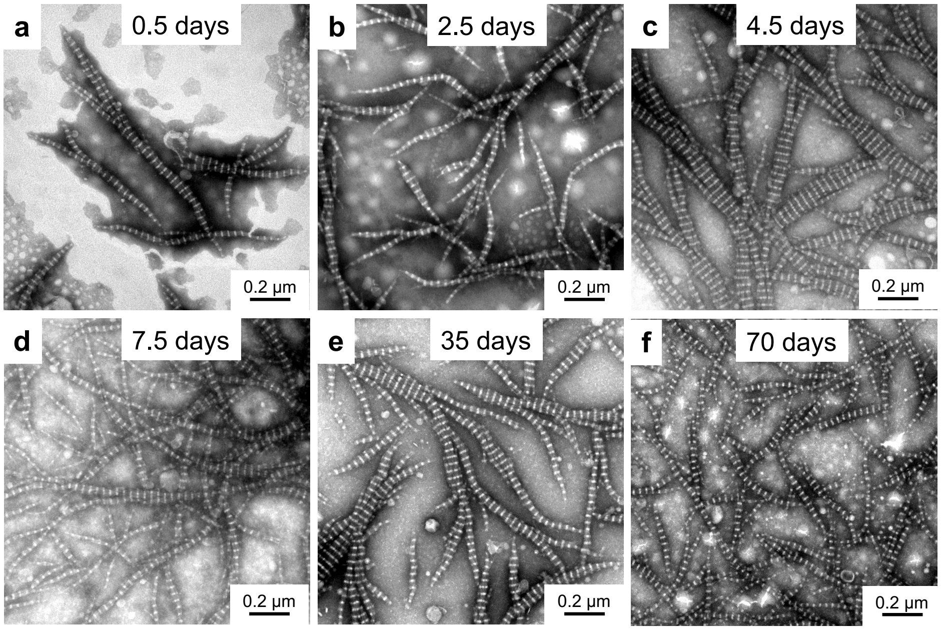

Supplementary Fig. 5. Transmission Electronic Microscopy (TEM) images of $P_{10}BP_{10}$ fibers self-assembled at various time. The samples prepared under the same condition as Fig. 1f, in a 10 mM phosphate buffer at pH 7 for 0.5 days **(a)**, 2.5 days **(b)**, 4.5 days **(c)**, 7.5 days **(d)**, 35 days **(e)**, 70 days **(f)**.

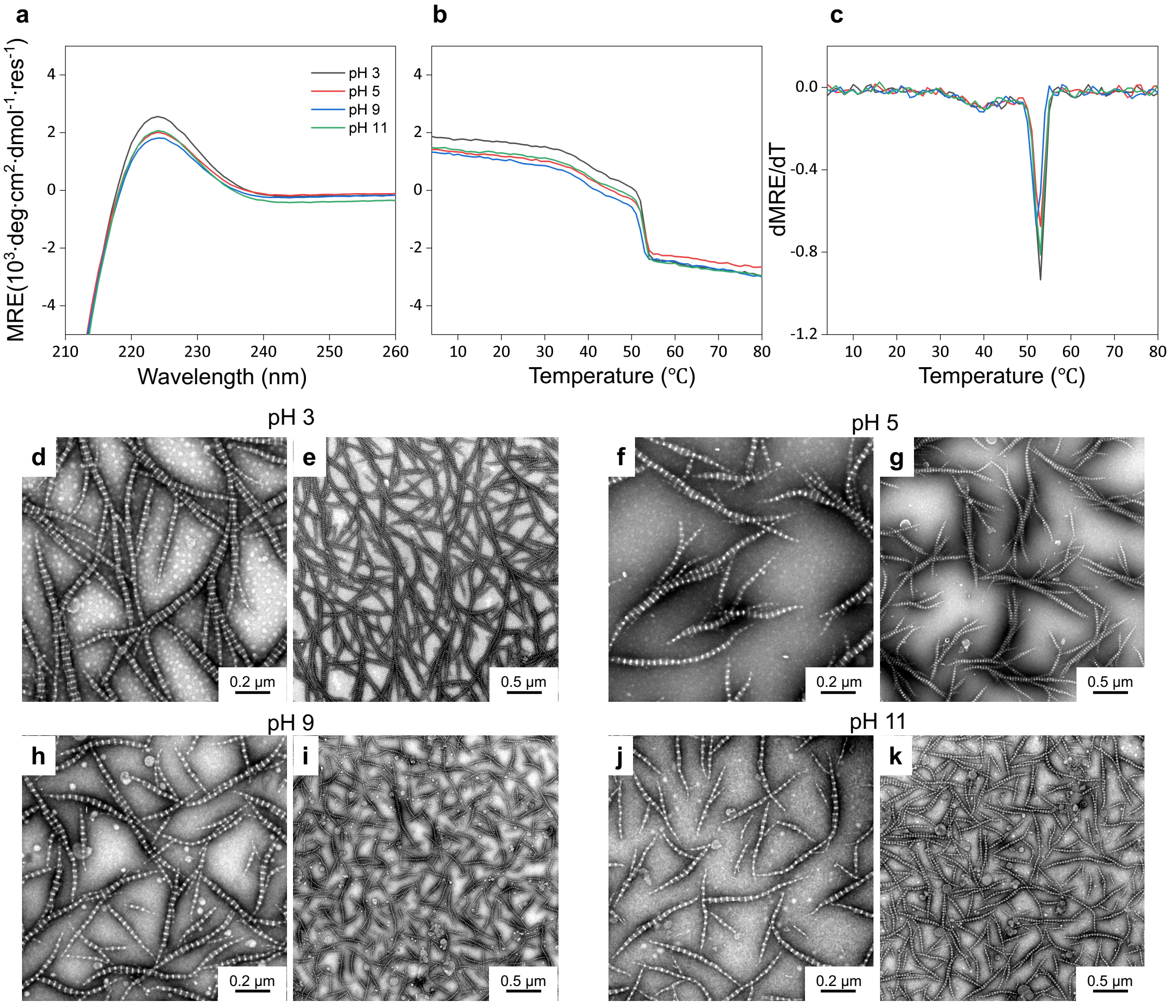

Supplementary Fig. 6. Circular dichroism characterization and Transmission Electronic Microscopy (TEM) images of $P_{10}BP_{10}$ fibers self-assembled at different pH. Wavelength scans **(a)**, thermal transitions curves **(b)**, and one order derivative of thermal transitions **(c)** of the samples. The samples prepared under the same condition as Fig. 1f, in a 10 mM phosphate buffer at pH 3 **(d, e)**, pH 5 **(f, g)**, pH 9 **(h, i)**, and pH 11 **(j, k)**, for 3 days.

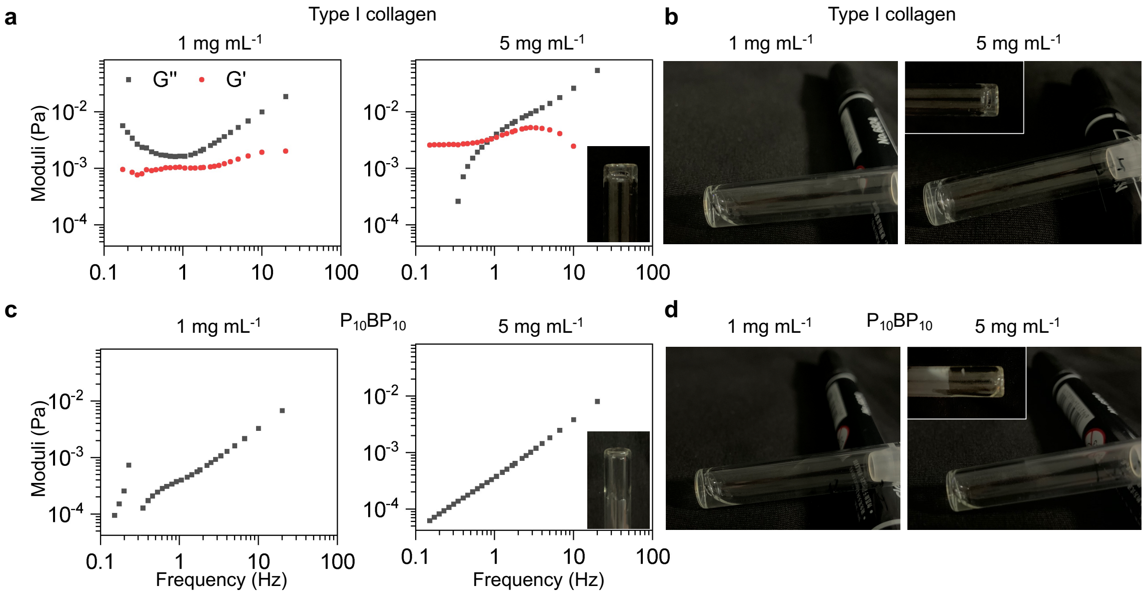

Supplementary Fig. 7. Microrheology storage and loss moduli, G′ and G″, and tilting tube test of the type I collagen **(a, b)** and $P_{10}BP_{10}$ **(c, d)** solutions at 1 and 5 mg mL^{-1} in 10 mM phosphate buffer (pH 7.4).

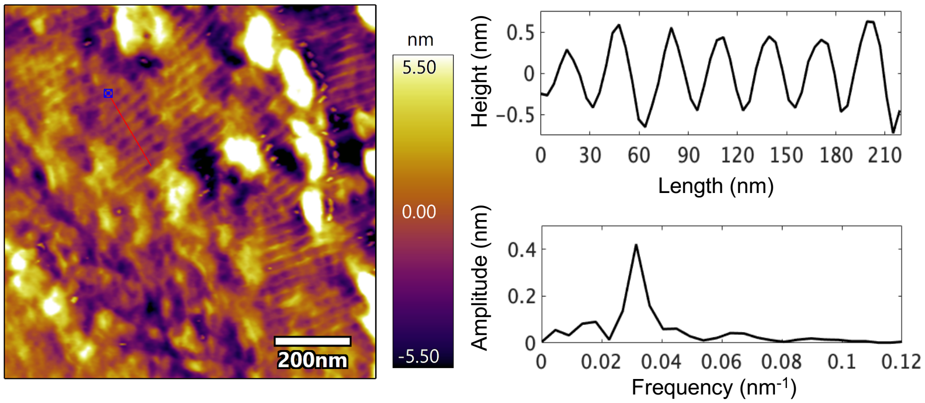

Supplementary Fig. 8. Atomic force microscopy (AFM) height scans of $P_{10}BP_{10}$ fibers and contour profiles of height. The peaks of frequencies after Fourier transformation show a uniform repeat frequency matching the protein length, and a height matching the diameter of a triple-helix.

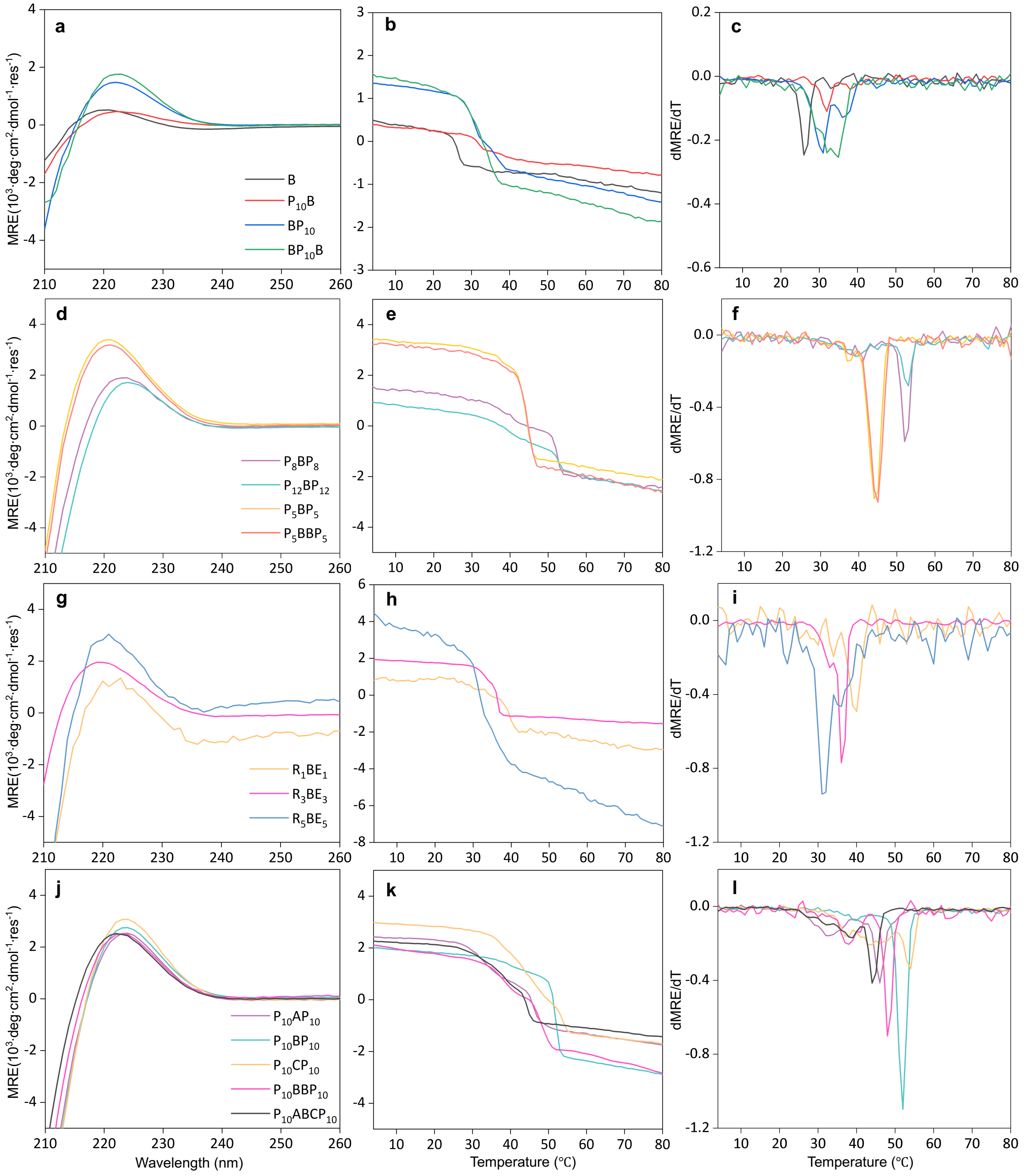

Supplementary Fig. 9. Circular dichroism characterization of the designed collagens in 10 mM PB at 4°C. (Left) wavelength scans, (Middle) thermal transitions curves, and (Right) one order derivative of thermal transitions of the samples. The sample names were as labeled.

Differential Scanning Calorimetry (DSC) were carried out on P_8BP_8, $P_{10}BP_{10}$, $P_{10}BBP_{10}$. Two peaks in DSC were consistent with two transitions in the melting curves in Circular Dichroism (Supplementary Fig.9). The narrow and sharp shape of the major peaks was resembled to those of bacteria collagen-like proteins, from which the functional-driver modules were derived, in previous study[1, 2]. The major peaks of the designed collagens were located between 50-60 °C, higher than those of the collagen-like proteins located between 30-40 °C[1, 2]. The increase in the melting temperatures could be resulted from both adding the Pro-rich adhesive modules to the two termini and formation of fibers. We would expect fibers to be stable under physiological conditions.

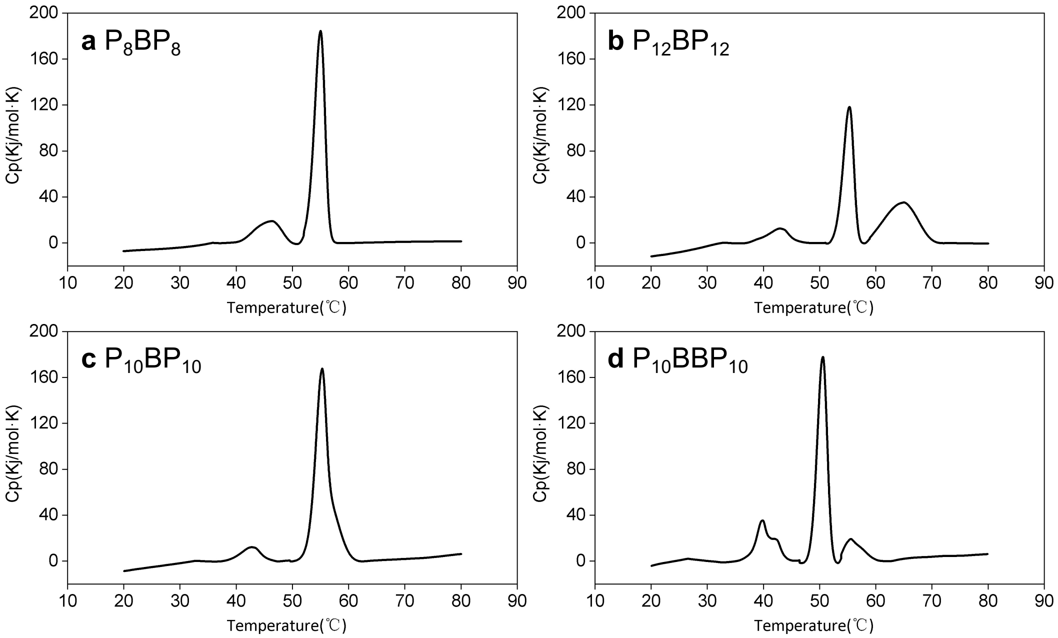

Supplementary Fig. 10. Differential scanning calorimetry of the synthetic collagen P_8BP_8 **(a)**, $P_{12}BP_{12}$ **(b)**, $P_{10}BP_{10}$ **(b)**, and $P_{10}BBP_{10}$ **(d)** at 5 mg mL^{-1} in 10 mM phosphate buffer (pH 7.4).

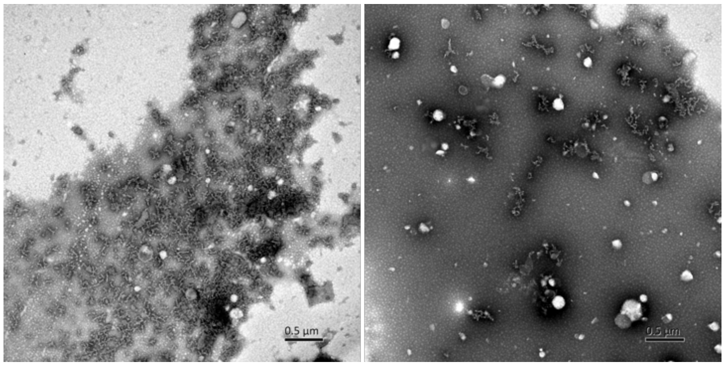

Supplementary Fig. 11. TEM images of $P_{10}BP_{10}$ (0.5 mM in 10 mM phosphate buffer at pH 7) after incubation at 70°C for 30 minutes and then self-assembled at 4°C for 3 days. The results showed that random aggregates formed after the triple helices were destroyed.

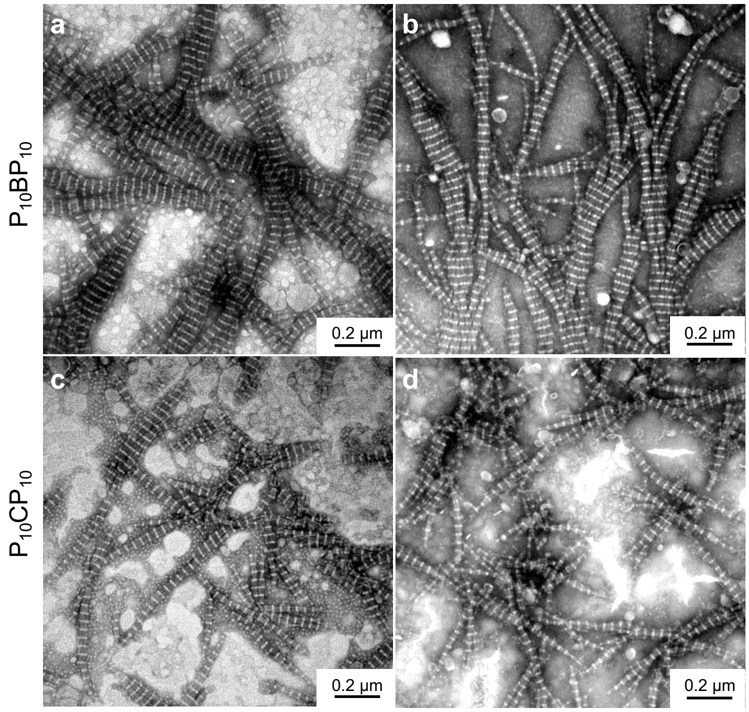

Supplementary Fig. 12. TEM images of $P_{10}BP_{10}$ **(a-b)**, and $P_{10}CP_{10}$ **(c-d)**, which had the same adhesive module but different functional-driver modules with various amino-acid composition. The samples were prepared under the same condition as Fig. 1f.

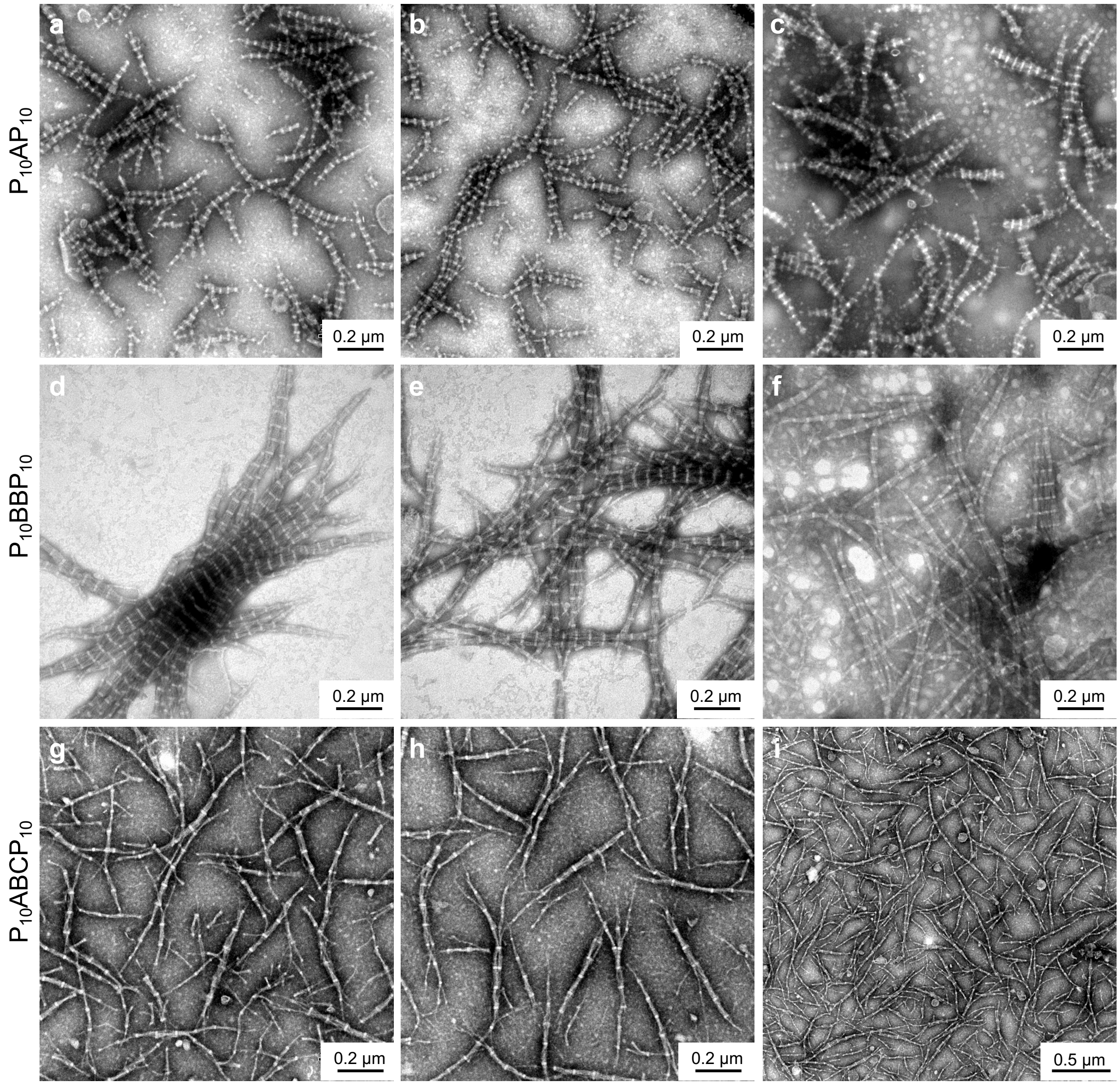

Supplementary Fig. 13. TEM images of the synthetic collagens, $P_{10}AP_{10}$ **(a-c)**, $P_{10}BBP_{10}$ **(d-f)** and $P_{10}ABCP_{10}$ **(g-i)**, which had the same adhesive module but the adhesive modules with various lengths.

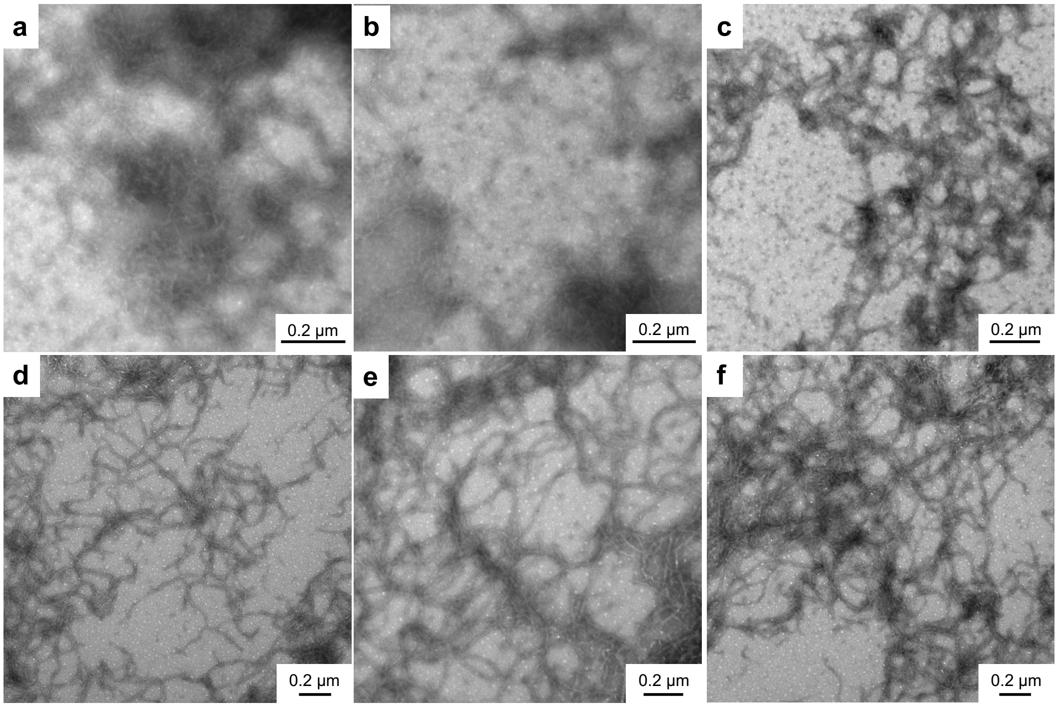

Supplementary Fig. 14. TEM images of the synthetic collagens, P_5BP_5 **(a-c)** and P_5BBP_5 **(d-f),** which formed fibers without any banding. The samples were prepared under the same condition as Fig. 1f.

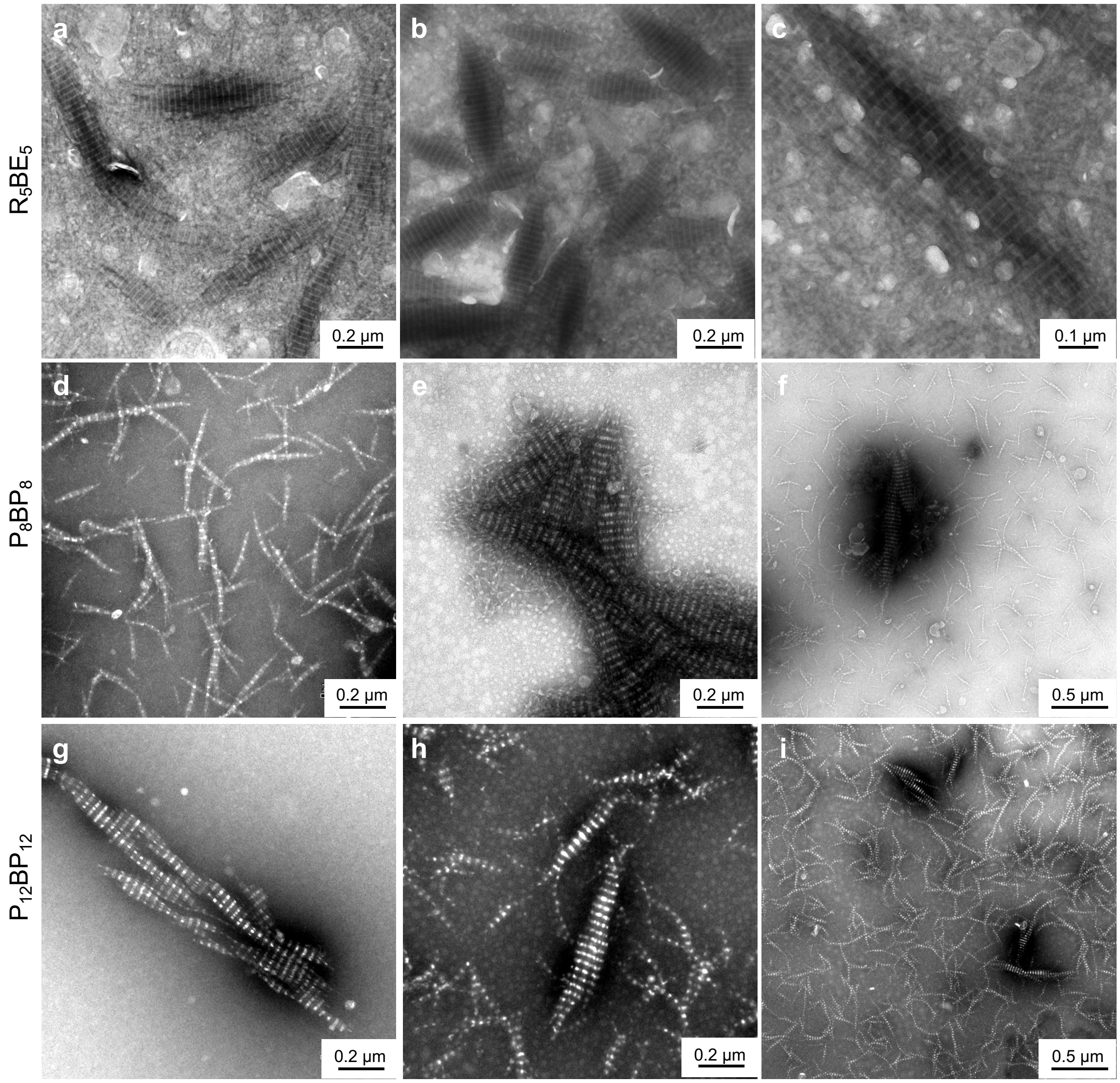

Supplementary Fig. 15. TEM images of the synthetic collagens, R_5BE_5 **(a-c)**, P_8BP_8 **(d-f)**, and $P_{12}BP_{12}$ **(g-i)**, which had the same functional-driver module but various adhesive modules. The samples were prepared under the same condition as Fig. 1f.

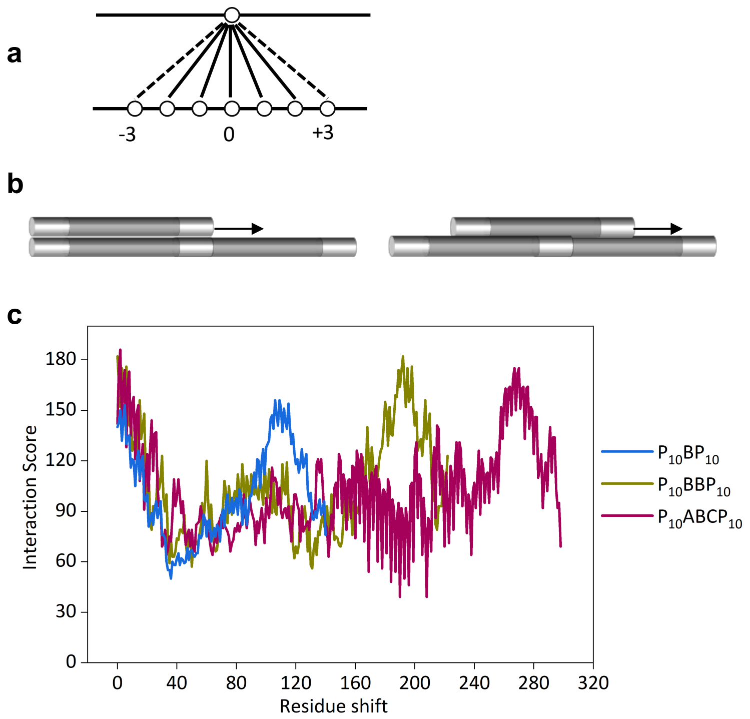

Supplementary Fig. 16. Prediction of inter-triple helical interactions. **a.** Interaction score were computed. When two charged residues are located within three residues, we count them as an electrostatic pair. When two hydrophobic residues are located within two residues, we count them as a hydrophobic pair. **b.** Schematic representation of inter-helical chain shifting of the synthetic collagens. The synthetic collagens, $P_{10}XP_{10}$, is composed of a functional-driver module, X, (X=B, BB, ABC) flanked by adhesive modules P_{10}. Note than only one adhesive model is shown between adjacent functional drivers to simulate the overlap of these regions in an assembling fiber. **c.** Interaction score vs. shifting length of $P_{10}XP_{10}$.

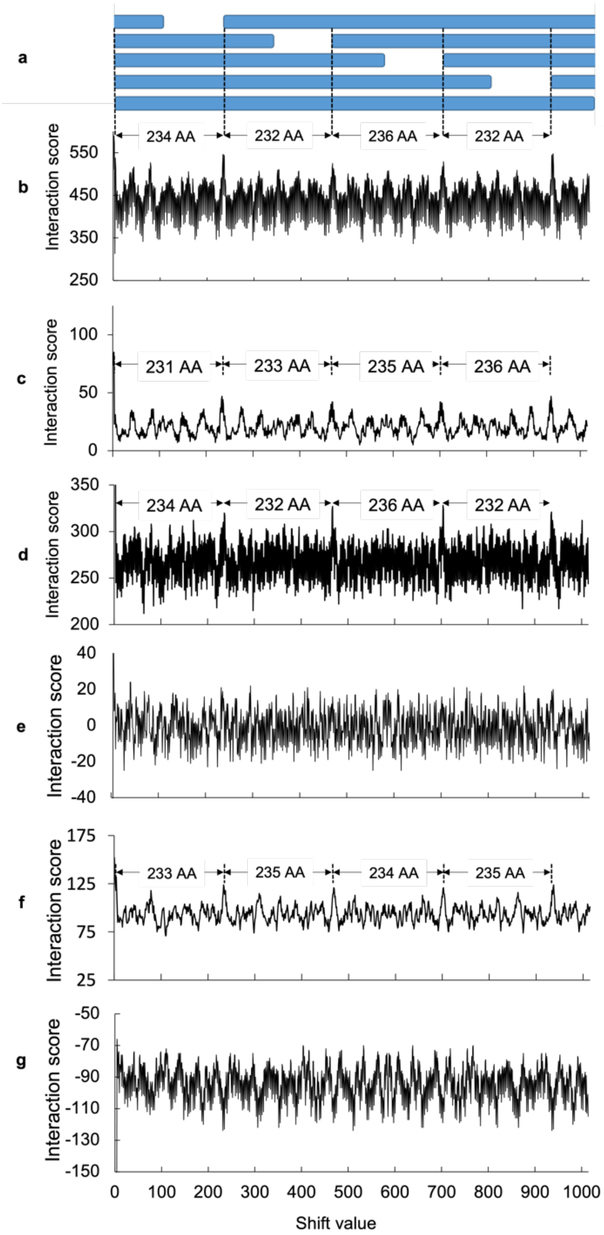

Supplementary Fig. 17. Interaction scores between two shifted sequences of α1 chains of natural Type I collagen. **a,** Schematic presentation of four shifted locations with a gap region, which are similar to the alignment of triple helices in Type I collagen, was aligned with the shifted residue numbers of *y* axis. The total calculated interaction showin in panel (**b**) is the sum of hydrophobic residues (Val, Ile, Leu, Phe, Met) **(c)** Pro **(d)** total electrostatics **(e)**. Electrostatic interactions are both attractive ion pairs **(f)** and repulsive (**g**). The residue numbers between large peaks of the interaction scores were as labeled in (b), (c) and (f).

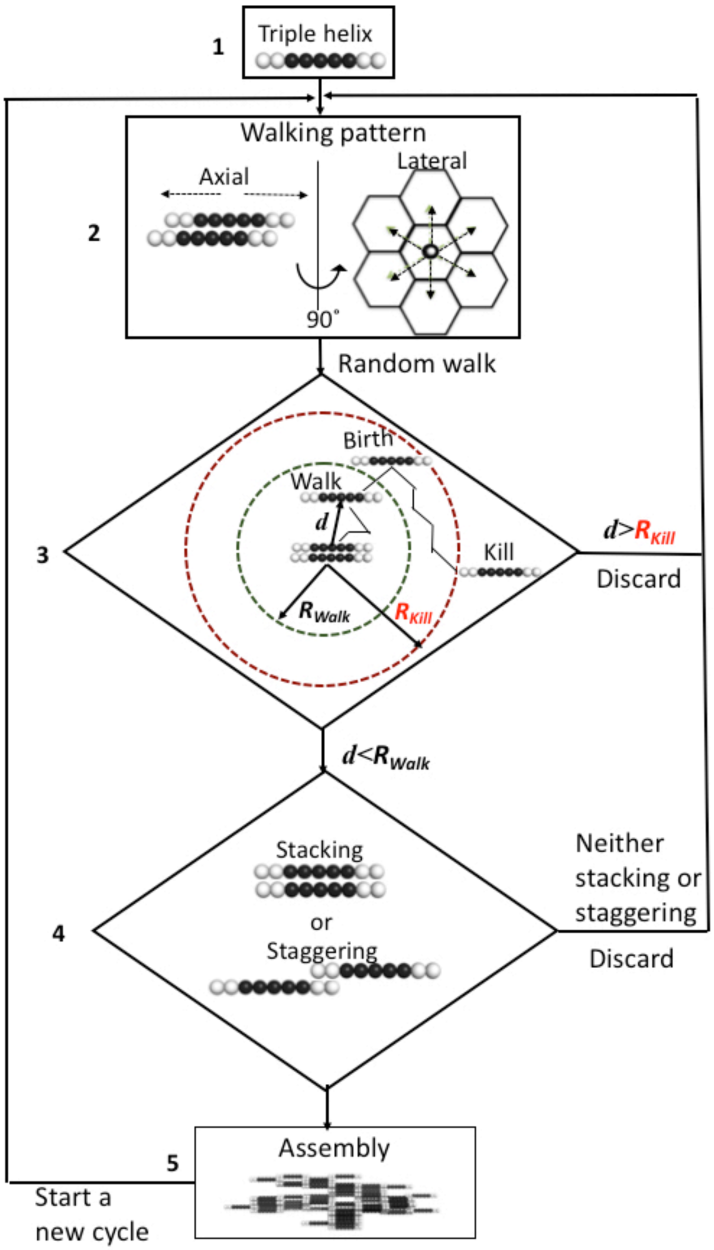

Supplementary Fig. 18. A flowchart of Diffusion Limited Aggregation (DLA), modified from previous study[3].

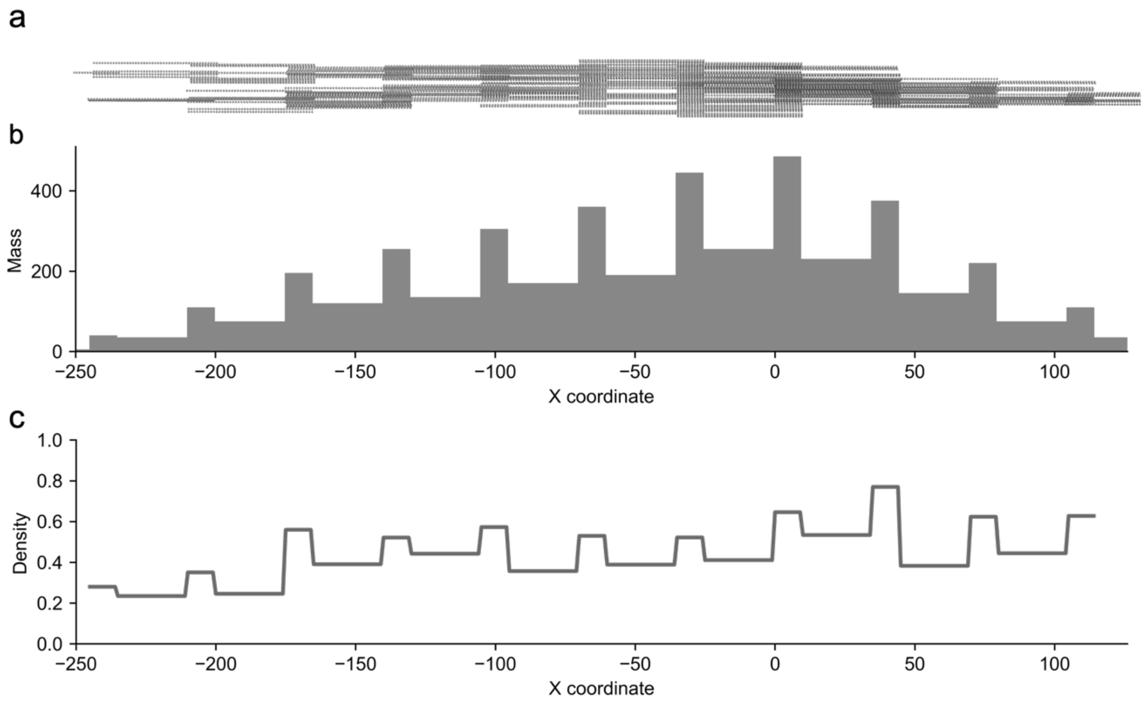

Supplementary Fig. 19. Diffusion-Limited Aggregation simulated fiber for $P_{10}BP_{10}$ (300 rods) (a), and the mass (b) and density (c) profiles along the fibril, showed that the adhesive modules formed regions with a high mass and density of spheres, while the functional-driver modules formed low mass and density regions. The number of spheres and the occupied volume for the diffusion-limited aggregation simulated fiber was statistic according to X coordinate. The mass of the diffusion-limited aggregation simulated fiber represent the number of spheres at each X coordinate. The density is the mass divided by the volume.

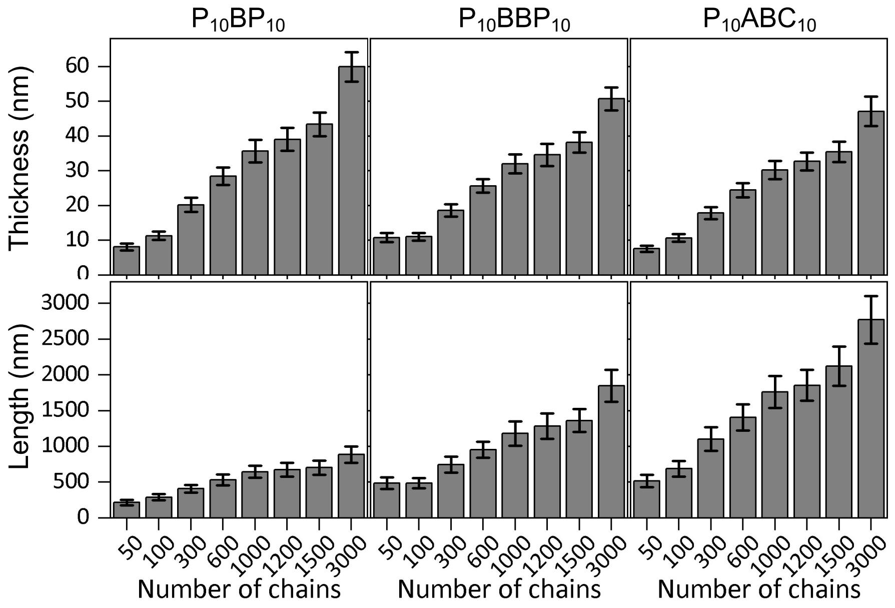

Supplementary Fig. 20. Thickness and length of the fibers simulated with Diffusion-Limited Aggregation (DLA) of the synthetic collagens, $P_{10}XP_{10}$ (X=B, BB, ABC), and the data are represented as the mean ± s.d. (n=100).

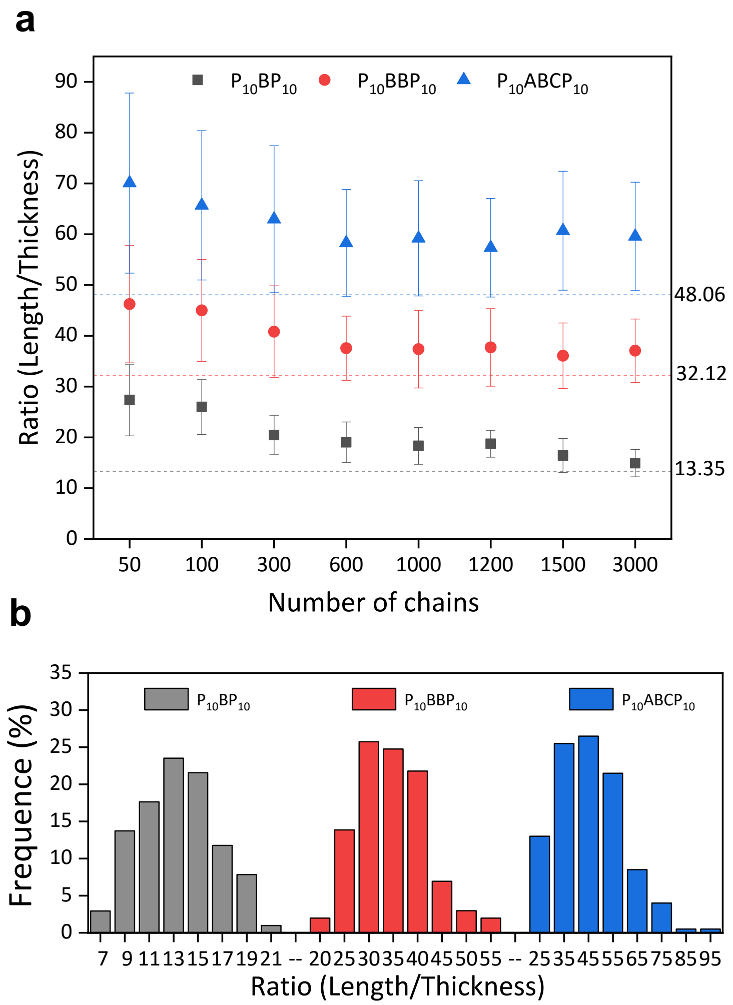

Supplementary Fig. 21. Comparison of length:thickness ratio of the fibers in DLA simulation vs. the TEM images (analyzed using ImageJ). (a) Length:thickness ratio was plotted against numbers of chains in the DLA simulation, and the data are represented as the mean ± s.d. (n = 100). Dashed lines indicated the average length:thickness ratios from the TEM images (analyzed using ImageJ). (b) The distribution histogram of length:thickness ratios from TEM images (n = 200).

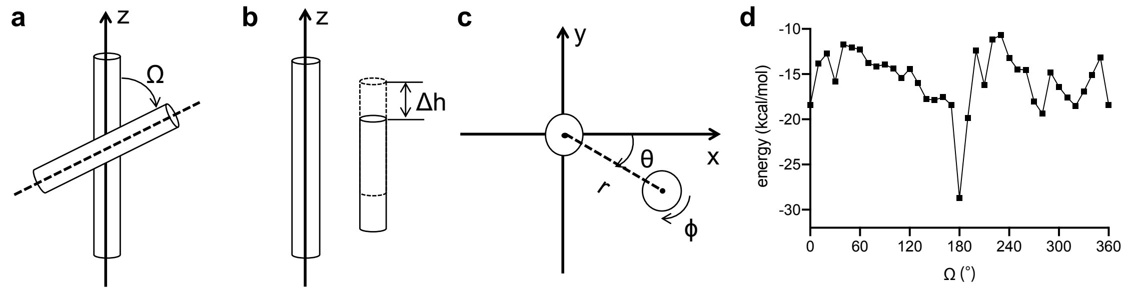

Supplementary Fig. 22. Geometric parameters describing inter-helical conformations of adhesive modules, modified from previous study[4]. **a,** The tilt angle of two triple helices (Ω); **b,** Translation along *z*-axis (Δh); **c,** Separation distance (r) and helical rotations (θ, φ); **d,** Interaction energy score of adhesive modules, $P_5 + P_5$, was plotted against Ω.

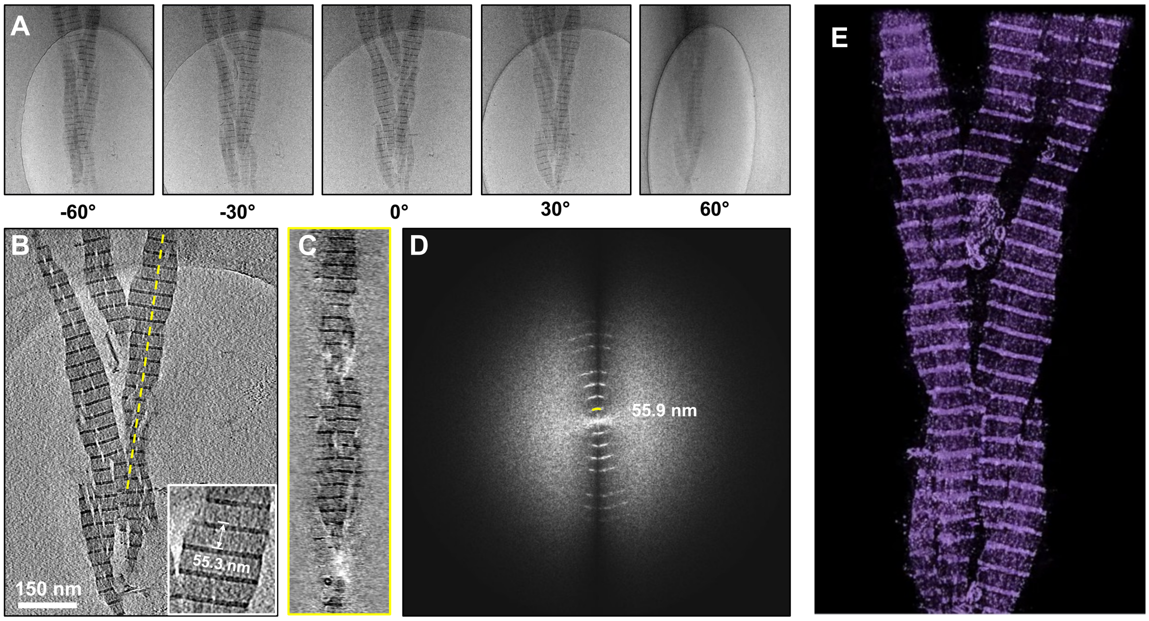

Supplementary Fig. 23. Cryo-electron tomography of $P_{10}BBP_{10}$ collagen fibrils. **a** Tilt series of collagen fibers at tilt angles ranging from -60° to +60°. **b** Slice views of the tomogram of $P_{10}BBP_{10}$ collagen fibers with characteristic banding pattern. Inset in (b) shows a zoomed-in view with measurement of periodicity. **c** Cross-section of a collagen bundle as indicated by a dashed yellow line in (b)**. d** Fourier transform of the image in (b) showing banding periodicity. **e** Volume rendering of collagen fibers showing dense overlap and sparse gap zones.

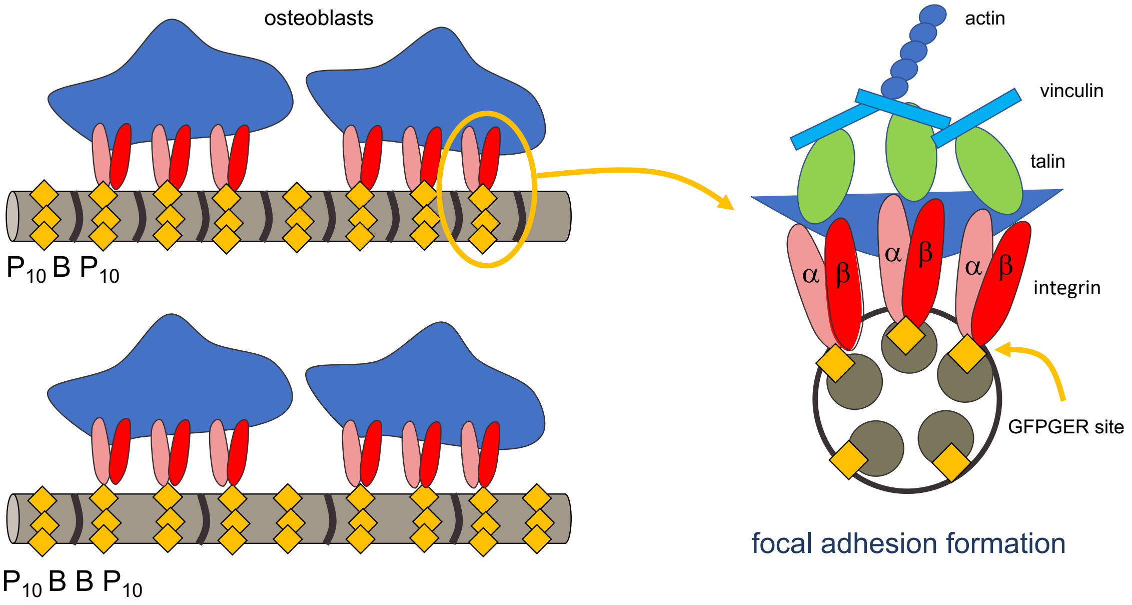

Supplementary Fig. 24. Hypothesized mechanism for banded synthetic collagen inducing osteoblastic differentiation. Both $P_{10}BP_{10}$ and $P_{10}BBP_{10}$ laterally align multiple GFPGER sites along the fiber axis. At each position, multiple integrins can bind, resulting in receptor clustering. This activates downstream signaling through factors such as talin and vinculin, and eventually driving cytoskeletal changes.

Supplementary Movie 1. Movie of slice views and volume rendering of $P_{10}BP_{10}$ collagen fibrils.

Supplementary Movie 2. Movie of slice views and volume rendering of $P_{10}BBP_{10}$ collagen fibrils.

Supplementary Table 1. List of full sequences of the synthetic collagens initially expressed in *E.coli*. The full sequences included a $(His)_6$ tag (HHHHHH), a globular domain, V, for folding, protease cleavage site (LVPRGSP), and a collagen domain. Sequence of the globular domain, highlighted in gray, is from *S.pyogenes* Scl2 gene. After removing the globular domain, the collagen domain remained and characterized, which was referred as the synthetic collagen.

V-$P_{10}AP_{10}$	HHHHHHADEQEEKAKVRTELIQELAQGLGGIEKKNFPTLGDEDLDHTYMT KLLTYLQEREQAENSWRKRLLKGIQDHALDLVPRGSPGPPGPPGPPGPPGP PGPPGPPGPPGPPGPPGQDGRNGERGEQGPTGPTGPAGPRGLQGLQGFPGE RGEQGPTGPAGPRGLQGERGEQGPTGLAGKAGEAGAKGETGPAGPQGPP GPPGPPGPPGPPGPPGPPGPPGPPG
V-$P_{10}BP_{10}$	HHHHHHADEQEEKAKVRTELIQELAQGLGGIEKKNFPTLGDEDLDHTYMT KLLTYLQEREQAENSWRKRLLKGIQDHALDLVPRGSPGPPGPPGPPGPPGP PGPPGPPGPPGPPGPPGPRGEQGPQGLPGKDGEAGAQGPAGPMGPAGFPGE RGEKGEPGTQGAKGDRGETGPVGPRGERGEAGPAGKDGERGPVGPAGPP GPPGPPGPPGPPGPPGPPGPPGPPG
V-P_8BP_8	HHHHHHADEQEEKAKVRTELIQELAQGLGGIEKKNFPTLGDEDLDHTYMT KLLTYLQEREQAENSWRKRLLKGIQDHALDLVPRGSPGPPGPPGPPGPPGP PGPPGPPGPPGPRGEQGPQGLPGKDGEAGAQGPAGPMGPAGFPGERGEKG EPGTQGAKGDRGETGPVGPRGERGEAGPAGKDGERGPVGPAGPPGPPGPP GPPGPPGPPGPPGPPG
V-$P_{12}BP_{12}$	HHHHHHADEQEEKAKVRTELIQELAQGLGGIEKKNFPTLGDEDLDHTYMT KLLTYLQEREQAENSWRKRLLKGIQDHALDLVPRGSPGPPGPPGPPGPPGP PGPPGPPGPPGPPGPPGPPGPPGPRGEQGPQGLPGKDGEAGAQGPAGPMGP AGFPGERGEKGEPGTQGAKGDRGETGPVGPRGERGEAGPAGKDGERGPV GPAGPPGPPGPPGPPGPPGPPGPPGPPGPPGPPGPPGPPG
V-$P_{10}CP_{10}$	HHHHHHADEQEEKAKVRTELIQELAQGLGGIEKKNFPTLGDEDLDHTYMT KLLTYLQEREQAENSWRKRLLKGIQDHALDLVPRGSPGPPGPPGPPGPPGP PGPPGPPGPPGPPGPPGKDGQNGQDGLPGKDGKDGQNGKDGLPGKDGKD GQNGKDGLPGKDGKDGQDGKDGLPGKDGKDGLPGKDGKDGQPGKPGPP GPPGPPGPPGPPGPPGPPGPPGPPG
V-$P_{10}BBP_{10}$	HHHHHHADEQEEKAKVRTELIQELAQGLGGIEKKNFPTLGDEDLDHTYMT KLLTYLQEREQAENSWRKRLLKGIQDHALDLVPRGSPGPPGPPGPPGPPGP

	PGPPGPPGPPGPPGPPGPRGEQGPQGLPGKDGEAGAQGPAGPMGPAGERGEKGEPGTQGAKGDRGETGPVGPRGERGEAGPAGKDGERGPVGPAGFPGERGPRGEQGPQGLPGKDGEAGAQGPAGPMGPAGERGEKGEPGTQGAKGDRGETGPVGPRGERGEAGPAGKDGERGPVGPAGPPGPPGPPGPPGPPGPPGPPGPPGPPG
V-P$_{10}$ABCP$_{10}$	HHHHHHADEQEEKAKVRTELIQELAQGLGGIEKKNFPTLGDEDLDHTYMTKLLTYLQEREQAENSWRKRLLKGIQDHALDLVPRGSPGPPGPPGPPGPPGPPGPPGPPGPPGPPGPPGQDGRNGERGEQGPTGPTGPAGPRGLQGLQGLQGERGEQGPTGPAGPRGLQGERGEQGPTGLAGKAGEAGAKGETGPAGPQGPRGEQGPQGLPGKDGEAGAQGPAGPMGPAGERGEKGFPGERGAKGDRGETGPVGPRGERGEAGPAGKDGERGPVGPAGKDGQNGQDGLPGKDGKDGQNGKDGLPGKDGKDGQNGKDGLPGKDGKDGQDGKDGLPGKDGKDGLPGKDGKDGQPGKPGPPGPPGPPGPPGPPGPPGPPGPPGPPGPPG
V-R$_{1}$BE$_{1}$	HHHHHHADEQEEKAKVRTELIQELAQGLGGIEKKNFPTLGDEDLDHTYMTKLLTYLQEREQAENSWRKRLLKGIQDHALDLVPRGSPGPPGPPGPRGPPGPPGPRGEQGPQGLPGKDGEAGAQGPAGPMGPAGFPGERGEKGEPGTQGAKGDRGETGPVGPRGERGEAGPAGKDGERGPVGPAGPPGPPGEPGPPGPPG
V-R$_{3}$BE$_{3}$	HHHHHHADEQEEKAKVRTELIQELAQGLGGIEKKNFPTLGDEDLDHTYMTKLLTYLQEREQAENSWRKRLLKGIQDHALDLVPRGSPGPPGPRGPRGPRGPPGPRGEQGPQGLPGKDGEAGAQGPAGPMGPAGFPGERGEKGEPGTQGAKGDRGETGPVGPRGERGEAGPAGKDGERGPVGPAGPPGEPGEPGEPGPPG
V-R$_{5}$BE$_{5}$	HHHHHHADEQEEKAKVRTELIQELAQGLGGIEKKNFPTLGDEDLDHTYMTKLLTYLQEREQAENSWRKRLLKGIQDHALDLVPRGSPGPRGPRGPRGPRGPRGPRGEQGPQGLPGKDGEAGAQGPAGPMGPAGFPGERGEKGEPGTQGAKGDRGETGPVGPRGERGEAGPAGKDGERGPVGPAGEPGEPGEPGEPGEPG
V-P$_{5}$BP$_{5}$	HHHHHHADEQEEKAKVRTELIQELAQGLGGIEKKNFPTLGDEDLDHTYMTKLLTYLQEREQAENSWRKRLLKGIQDHALDLVPRGSPGPPGPPGPPGPPGPPGPRGEQGPQGLPGKDGEAGAQGPAGPMGPAGFPGERGEKGEPGTQGAKGDRGETGPVGPRGERGEAGPAGKDGERGPVGPAGPPGPPGPPGPPGPPG
V-P$_{5}$BBP$_{5}$	HHHHHHADEQEEKAKVRTELIQELAQGLGGIEKKNFPTLGDEDLDHTYMTKLLTYLQEREQAENSWRKRLLKGIQDHALDLVPRGSPGPPGPPGPPGPPGPPGPRGEQGPQGLPGKDGEAGAQGPAGPMGPAGFPGERGEKGEPGTQGAKGDRGETGPVGPRGERGEAGPAGKDGERGPVGPAGPRGEQGPQGLPGKDGEAGAQGPAGPMGPAGFPGERGEKGEPGTQGAKGDRGETGPVGPRGERGEAGPAGKDGERGPVGPAGPPGPPGPPGPPGPPG
V-B	HHHHHHADEQEEKAKVRTELIQELAQGLGGIEKKNFPTLGDEDLDHTYMTKLLTYLQEREQAENSWRKRLLKGIQDHALDLVPRGSPGPRGEQGPQGLPGKDGEAGAQGPAGPMGPAGFPGERGEKGEPGTQGAKGDRGETGPVGPRGERGEAGPAGKDGERGPVGPAG

V-P_{10}B	HHHHHHADEQEEKAKVRTELIQELAQGLGGIEKKNFPTLGDEDLDHTYMTKLLTYLQEREQAENSWRKRLLKGIQDHALDLVPRGSPGPPGPPGPPGPPGPPGPPGPPGPPGPPGPPGPRGEQGPQGLPGKDGEAGAQGPAGPMGPAGFPGERGEKGEPGTQGAKGDRGETGPVGPRGERGEAGPAGKDGERGPVGPAG
V-BP_{10}	HHHHHHADEQEEKAKVRTELIQELAQGLGGIEKKNFPTLGDEDLDHTYMTKLLTYLQEREQAENSWRKRLLKGIQDHALDLVPRGSPGPRGEQGPQGLPGKDGEAGAQGPAGPMGPAGFPGERGEKGEPGTQGAKGDRGETGPVGPRGERGEAGPAGKDGERGPVGPAGPPGPPGPPGPPGPPGPPGPPGPPGPPGPPG
V-BP_{10}B	HHHHHHADEQEEKAKVRTELIQELAQGLGGIEKKNFPTLGDEDLDHTYMTKLLTYLQEREQAENSWRKRLLKGIQDHALDLVPRGSPGPRGEQGPQGLPGKDGEAGAQGPAGPMGPAGFPGERGEKGEPGPPGPPGPPGPPGPPGPPGPPGPPGPPGPPGTQGAKGDRGETGPVGPRGERGEAGPAGKDGERGPVGPAG

Supplementary Table 2. The geometric parameters of the parallel ($\Omega = 0°$) and antiparallel ($\Omega = 180°$) interhelical association of the adhesive modules between Pn + Pn and Rn + En.

	Sequence	Ω (°)	ϕ (°)	θ (°)	r (Å)	Δh (Å)	Energy
Anti-parallel	P_5BP_5	180	−48.8	31.2	11.6	1.6	−29.5
	P_8BP_8	180	−50.8	25.8	11.8	1.7	−46.1
	$P_{10}BP_{10}$	180	−47.7	31.8	11.7	1.5	−55.0
	$P_{12}BP_{12}$	180	−52.5	26.2	11.9	1.5	−67.7
	R_1BE_1	180	−39.7	37.9	11.6	1.9	−12.8
	R_3BE_3	180	34.4	−4.3	14.2	−1.5	−25.7
	R_5BE_5	180	−8.2	−41.2	13.4	−1.7	−37.9
Parallel	P_5BP_5	0	−48.2	29.4	12.2	−0.9	−14.8
	P_8BP_8	0	−49.5	27.0	12.1	−1.0	−27.2
	$P_{10}BP_{10}$	0	−45.5	31.5	12.1	−1.0	−31.3
	$P_{12}BP_{12}$	0	−45.5	31.8	12.5	−0.7	−35.9
	R_1BE_1	0	−6.4	40.2	11.6	−0.8	−18.4
	R_3BE_3	0	29.4	21.1	13.1	2.1	−29.9
	R_5BE_5	0	38.8	30.3	13.3	2.0	−47.6

Supplementary Table 3. Primers used for Q-PCR to measure the expression level of adhesion and osteogenic differentiation related genes.

Primers	Sequence (5'-3')
Mouse Gapdh F	AGGTCGGTGTGAACGGATTTG
Mouse Gapdh R	TGTAGACCATGTAGTTGAGGTCA
Mouse Vinculin F	CTCGTCCGGGTTGGAAAAGAG
Mouse Vinculin R	AGTAAGGGTCTGACTGAAGCAT
Mouse Actn F	TCCATCGGAGCCGAAGAAATC

Mouse Actn R	GTGTCGGTGGATCAAAGCACA
Mouse Runx2 F	GCACAAACATGGCCAGATTCA
Mouse Runx2 R	AAGCCATGGTGCCCGTTAG

Supplementary References:

1. Yoshizumi, A. et al. Self-association of streptococcus pyogenes collagen-like constructs into higher order structures. *Protein Science* **18**, 1241-1251 (2009).
2. Hu, J. et al. Design Strategies to Tune the Structural and Mechanical Properties of Synthetic Collagen Hydrogels. *Biomacromolecules* **22**, 3440-3450 (2021).
3. McGuinness, K., Khan, I.J. & Nanda, V. Morphological diversity and polymorphism of self-assembling collagen peptides controlled by length of hydrophobic domains. *Acs Nano* **8**, 12514-12523 (2014).
4. Xu, F. et al. Self-Assembly of Left- and Right-Handed Molecular Screws. *Journal of the American Chemical Society* **135**, 18762-18765 (2013).
