# Supplementary material for: Design of synthetic collagens that assemble into supramolecular banded fibers as a functional biomaterial testbed
Source: Nat Commun. 2022 Nov 9;13:6761. doi: 10.1038/s41467-022-34127-6 (PMC9646729; doi:10.1038/s41467-022-34127-6)
Supplement: Supplementary file 3 — Description of Additional Supplementary Files [file 41467_2022_34127_MOESM3_ESM.pdf]

Description of Additional Supplementary Files

File Name: Supplementary Movie 1
Description: Movie of $P_{10}BP_{10}$ cryo-ET tomograms showing the tilt series, slice views and volume rendering.

File Name: Supplementary Movie 2
Description: Movie of $P_{10}BBP_{10}$ cryo-ET tomograms showing the tilt series, slice views and volume rendering.
